# Soft Sensor Application in Identification of the Activated Sludge Bulking Considering the Technological and Economical Aspects of Smart Systems Functioning

**DOI:** 10.3390/s20071941

**Published:** 2020-03-30

**Authors:** Bartosz Szeląg, Jakub Drewnowski, Grzegorz Łagód, Dariusz Majerek, Ewa Dacewicz, Francesco Fatone

**Affiliations:** 1 Faculty of Environmental, Geomatic and Energy Engineering, Kielce University of Technology, Tysiąclecia Państwa Polskiego 7, 25-314 Kielce, Poland; 2 Faculty of Civil and Environmental Engineering, Gdansk University of Technology, Narutowicza 11/12, 80-233 Gdansk, Poland; jdrewnow@pg.edu.pl; 3 Faculty of Environmental Engineering, Lublin University of Technology, Nadbystrzycka 40B, 20-618 Lublin, Poland; 4 Faculty of Fundamentals of Technology, Lublin University of Technology, Nadbystrzycka 38, 20-618 Lublin, Poland; d.majerek@pollub.pl; 5 Faculty of Environmental Engineering and Land Surveying, University of Agriculture in Krakow, Mickiewicza 24/28, 30-059 Kraków, Poland; ewa.wasik@urk.edu.pl; 6 Department of Science and Engineering of Materials, Environment and Urban Planning-SIMAU, Polytechnic University of Marche Ancona, 60121 Ancona, Italy; f.fatone@univpm.it

**Keywords:** soft sensor, smart systems and infrastructure, data mining, classification model, wastewater treatment plant, activated sludge bulking

## Abstract

The paper presented the methodology for the construction of a soft sensor used for activated sludge bulking identification. Devising such solutions fits within the current trends and development of a smart system and infrastructure within smart cities. In order to optimize the selection of the data-mining method depending on the data collected within a wastewater treatment plant (WWTP), a number of methods were considered, including: artificial neural networks, support vector machines, random forests, boosted trees, and logistic regression. The analysis conducted sought the combinations of independent variables for which the devised soft sensor is characterized with high accuracy and at a relatively low cost of determination. With the measurement results pertaining to the quantity and quality of wastewater as well as the temperature in the activated sludge chambers, a good fit can be achieved with the boosted trees method. In order to simplify the selection of an optimal method for the identification of activated sludge bulking depending on the model requirements and the data collected within the WWTP, an original system of weight estimation was proposed, enabling a reduction in the number of independent variables in a model—quantity and quality of wastewater, operational parameters, and the cost of conducting measurements.

## 1. Introduction

The processes occurring in the environment, including urban areas, are very difficult to describe. These changes are dynamic in nature and are governed by a number of external factors, that are random (weather conditions), anthropogenic (traffic volume, amount of water supplied to the network) and local. Taking into account that many of them affect the operational costs of infrastructure, urban development, and the comfort of city residents, it is necessary to predict, control and optimize these factors [1,2]. This approach results in the development of the so-called smart system and infrastructure within smart cities [3,4,5]. Due to the complex process mechanisms and the influence of numerous factors, great quantities of data have to be collected using sensors. This enables us to identify their dynamics and create so-called soft sensors based on the devised the mathematical models [6,7,8]. In numerous cases, this solution enables us to reduce the amount of collected data as well as identify the phenomena, the time course of which is difficult to measure and, for example, generates high operational costs of the measurement system. The approach involving the creation of soft sensors supporting infrastructure is increasingly popular, especially in large cities which require their on-line control and optimization [9], as well as in such technological objects as wastewater treatment plants (WWTPs). The concept and implementation of soft sensors at WWTPs is well established, because they are employed for simulating the complex processes occurring in bioreactors [10]. Soft sensors were used for predicting total nitrogen at the WWTP outlet on the basis of the influent wastewater quality and operational parameters; this enables on-line process control [11]. Another soft sensor concept was also devised for this purpose, where the content of nitrogen compounds was modeled at the outlet based on the primary settling tank operation, internal recirculation, and wastewater temperature parameters [8]. A much more complex soft sensor concept was presented by Canete et al. [12], who predicted the chemical oxygen demand (COD), total suspended solids (TSS), and total nitrogen (TN) values at the WWTP outlet using artificial neural networks. The results of calculations and the settings were verified through the simulations conducted by means of a mechanistic calibrated WWTP model. Luccarni et al. [13] presented the concept of a soft sensor used for N-NH_4_ prediction in bioreactor chambers based on pH, oxidation-reduction potential (ORP), and dissolved oxygen concentration in the bioreactor (DO), which obtained satisfactory calculation results. Hernández-del-Olm et al. [14], taking into account the high cost of calculating important quality markers at the plant inlet (COD, ammonium nitrogen (N–NH_4_)) proposed a soft sensor for predicting their value for dry-weather and wet-weather as well as intense rainfall events. The concept of soft sensor for BOD prediction at the WWTP outlet was presented by Yu et al. [15], who employed the learning machine model for its creation, in which the values of the weights obtained were optimized by means of the cuckoo search algorithm. When assessing the processes conducted in treatment plant devices and the quality of treated wastewater, soft sensors are used to analyze multidimensional data sets obtained with gas sensor arrays [16,17,18,19]. At present, attention is also increasingly drawn to the energy aspects at the stage of optimization and control of technological processes, which is reflected in numerous papers on this topic [20,21,22].

Activated sludge sedimentation is one of the essential processes affecting the operation of biological reactors, specifically their efficiency. In WWTPs, it is assessed in terms of the sludge volumetric index (SVI). The parameter enables control and optimization of a bioreactor operation, thus affecting the management of the facility. In the WWTPs with integrated removal of carbon, nitrogen and phosphorus compounds, the recommended maximum SVI is 150 cm^3^/g [23]. If the value is exceeded, problems may occur with sludge dewatering and with the deteriorated quality of the discharged water. In order to keep the value of SVI within the optimal range, a mathematical model which enables the dynamic control of the SVI by proper selection of settings and a balanced management of the WWTP, must be constructed. On the one hand, the mathematical model must accurately reflect the course of a phenomenon, and on the other, its implementation should not be very complicated. Moreover, the number of the exogenous variables should be as small as possible.

The literature survey [24,25,26] indicates that a predominant majority of the models used for the SVI simulations are those predicting continuous values. These are usually data-mining methods, specifically artificial neural network methods and their complex modifications. Building complex model structures improves predictive power in the presently-known models and reduces the number of independent variables in models [26,27,28], but it also may lead to management problems. In models based on conventional methods, the number of independent variables is usually higher than that in complex models. The literature review [29,30,31,32,33,34,35,36,37,38] indicates that the classification models are built using a number of statistical methods, ranging from analytical (logistic regression) through more modified models employing the concept of regression trees (BT—boosted trees, RF—random forests), to neural networks. In the case of classification models, the basis for the identification of the bulking process involves the limit SVI values indicating problems with sedimentation.

At the stage of model creation, it is necessary to find a balance between the model complexity and its accuracy. The conducted analyses [39,40,41,42,43,44,45,46,47,48] showed that in numerous cases, modification of the RF and BT method leads to an improvement in the prediction capability of the regression tree model. This confirms the essential effect of implementing the boosting method in BT and substituting a single tree with a forest in the RF method. In the case of unsatisfactory simulation results, more complex methods than BT and RF can be employed, i.e., artificial neural networks. The model structure is many times more complex than in the methods mentioned above.

According to [12,49,50,51,52,53] the multi-layer perceptron (MLP) is one of the most frequently used methods. It comprises three layers: the input, the hidden, and the output layers. In this method, the so-called learning step identifies the values of what is called “weight”, which connect the neurons in the consecutive layers. In that method, the initial weight factor values have an essential effect on the course of the learning process. This may affect the predictive power of a model, and the results obtained using RF and BT may even be better than those provided by MLP. The shortcomings of MLP were eliminated in the support vector achine (SVM) method [54,55]. In the case of a non-linear relationship between the model output (y) and explanatory variables (x_1_, x_2_, …, x_n_) transformation of n-dimensional space to K-dimensional (linear) space of variable features is performed using the kernel function. Vapnik [55] has developed a special learning algorithm in the support vector method. Vapnik identified the scale values by solving the problem of square programming, which guarantees the existence of a single minimum. Consequently, at the operating and management levels (intended to obtain the optimum SVI by selecting the correct settings to limit problems with the continuity of processes), using that model for the monitoring and correcting sludge sedimentation is a source of technical problems. Constructing the models with a significant number of independent variables requires a constant monitoring of the values of operating parameters and numerous indicators of the wastewater quality; at the operating level, this creates problems with the process control and management. Obtaining large amounts of data with high resolution under working conditions is difficult for a number of reasons. In order to provide reliable input data for a model, the analyzing equipment must be calibrated on an ongoing basis. Moreover, the hardware sensors are prone to failure or sustaining mechanical damage under real conditions. Although it is still feasible to eliminate such damage or to calibrate the equipment as frequently as necessary, these tasks may be quite expensive and the costs of their implementation under operating and management conditions in a real facility may be disproportionately high. In the facilities operated with no hardware sensors, the indicators of quality and the bioreactor operating parameters are measured with analytical methods. From the technical point of view, obtaining data in a continuous system in that case is rather difficult. Therefore, the implementation of a calculation model in a WWTP may be limited, thus complicating the management of the facility. Despite the many problems with data acquisition, with their reliability and with the costs of measurements, researchers [56,57] still strive to improve the predictive power of models. The approach calls for some criticism, not being fully consistent with the latest trends, as these tend to focus on cost minimization [58,59,60,61]. On the other hand, systems that enable an on-line process control, taking into account the potential failures of measurement schemes, are required. In order to solve this problem, researchers will create new standards for the construction of mathematical models for controlling technological processes in wastewater treatment plants.

Considering the aforementioned observations, the different conditions and data acquisition standards applicable to WWTPs, it seems desirable to seek systems enabling process simulation with high accuracy, taking into account the real-time differences in the operation of WWTP facilities. Therefore, it seems reasonable to aim at such solutions for the simulation of the wastewater treatment processes that enable the processes to be controlled and operation of the facility to be managed regardless of the measured data (including the wastewater quality, operating parameters and their combinations). On the other hand, a process engineer working in a WWTP will be interested in selecting such independent variables that make the model accurate, even though the number of variables included in the model is limited: less measured data will generate lower operating costs but will still enable control of the wastewater treatment process. These assumptions can be implemented under operating conditions and the choice of the simulation method is of major importance. On the one hand, the calculation model must provide a compromise between complexity and accuracy. On the other hand, the question arises whether a model must be created to simulate continuous values. By introducing binary input/output variables, in many cases it is possible to obtain the models in the form of analytical dependencies, which can be applied without the need to implement complex computational algorithms.

As part of the analyses, the concept of a soft sensor system expert system has been proposed which can be applied in selecting a method for the analysis of activated sludge bulking and which takes into account the economic aspects of the wastewater treatment technology. These include the measured indicators of the wastewater quality, the operating parameters of the bioreactor, the duration of the test period, the influence of measurement errors of several independent variables on the results of a simulation, and the complexity of the method used in building the model for soft sensor construction.

## 2. Materials and Methods

### 2.1. Experimental Data

Experimental data were obtained from the test facility the WWTP in Sitkówka-Nowiny, located near the city of Kielce in the south of Poland. The daily load of the test facility is Q_n_ = 42,000 m^3^/d (equivalent to 2,750,000 PE) of municipal wastewater from Kielce and the adjacent area. The wastewater is first handled mechanically on bar screens, in a preliminary sedimentation tank basin and a sand trap. It is then sent downstream for biological treatment in a bioreactor, operated in a modified BARDENPHO system with a pre-denitrification chamber. The treated wastewater is separated from the activated sludge in the secondary sedimentation tank and is then discharged to the receiving body—the Silnica river.

Monitoring in the WWTP includes measurements of the quantity and quality of the influent wastewater and the bioreactor operating parameters. During the research period, a qualitative analysis of both the influent and effluent wastewater was performed once a week to determine its BOD, COD, TSS, TN, TP, N–NH4, and sedimentation parameters of activated sludge (SVI). The organic compounds were determined as COD in accordance with PN–ISO 6060: 2006 and as BOD with the method using the OXITOP, in accordance with PN–EN 1899–1:2002. TSS were determined by means of glass fiber filters with the methods set out in PN–EN 872: 2007. TP was determined in accordance with PN–EN ISO 6878: 2006. TN and nitrogen as N–NH4 were determined in accordance with PN–C–04576–14: 1973 and PN–ISO 5664: 2002, respectively. SVI was determined by means of the method set out in PN–EN 14702–1: 2008. Moreover, the quantity of the influent wastewater and the operating parameters of the reactor were measured, whereas the data were recorded at hourly intervals using SCADA. In this way, 250 datasets representing all the above mentioned variables were obtained. The monitored parameters included MLSS—mixed liquor suspended solids concentration of activated sludge, T—temperature in the bioreactor, DO—oxygen level, m_PIX_—dosage of PIX chemical coagulant, WAS—excess sludge, RAS—degree of recirculation [return activated sludge], and MLSSR—concentration of return sludge. The results of measurements in the various seasons of the years 2013–2015 are shown in Table 1. These data show that both the bioreactor operating parameters as well as the amount and quality of wastewater varied considerably in the different seasons of the study period, affecting the activated sludge sedimentation to a large extent. In the winter season, the average values of SVI were higher than those from the spring to fall. The relation obtained is confirmed by analysis of variance (ANOVA) test results. For the assumed value of p = 0.05, p-test value equal to p = 0.000024 and F = 42.257 were obtained. The SVI lability depending on temperature may indicate that the activated sludge bulking occurred in the period of interest due to a temperature drop, as confirmed in the analyses reported by Bayo et al. [62]. Other reasons could include a drop in the concentration of organic compounds in the influent wastewater as well as the changes in the oxygen concentration in the activated sludge chambers, as suggested by Comas et al. [29].

These results and the wide range of SVI indicates the requirement to build a mathematical model enabling the construction of soft sensor to control of the process of activated sludge sedimentation.

The literature [23,24,25,26] shows that the sludge bulking is a complex phenomenon. Its course and dynamics are influenced by temperature and pH, because they determine the growth of microorganisms. An increase in temperature raises the number of the floc-forming bacteria. The pH value should range between 7–7.5, since a drop below 6.0 results in the growth of fungi, which leads to sludge bulking. The MLSS and TSS values are the factors describing the amount of activated sludge in a bioreactor. The wastewater quality markers (BOD, COD, TN, TP, N-NH_4_) are a source of nutrients for microorganisms. Insufficient TN and TP lead to the creation of particles with high floc content and loss of sedimentation ability. The amount of coagulant usually leads to an improvement in the sedimentation of the activated sludge. Oxygen is used during obtaining energy for the biochemical processes conducted by microorganisms in the bioreactor.

### 2.2. Model Concept

**Figure 1:**
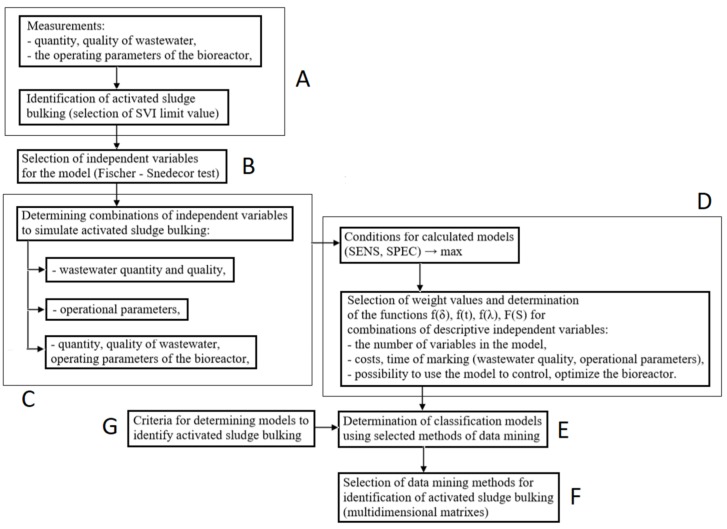
A schematic of the soft sensor system for selecting a data mining method for simulating sludge bulking.

This paper presents a concept of soft sensor for selecting the data-mining method for simulating the activated sludge bulking (or absence of bulking) which takes into account the following aspects: complexity of the data-mining method, time of analysis of independent variables in these simulations, different accessibilities of the recorded measurement data within the test facility, reliability of the measured data, usefulness of the model in process control and optimization (Figure 1). The approach presented below also indicates the possibility of extending the soft sensor system, to select the calculation method for various volumes and qualities of the influent wastewater and for various weather conditions.

In the approach presented, the model for assessing the activated sludge sedimentation was built based on the technological data recorded within the test facility. The various categories of technological data were determined by the type of the measurement system used by the test facility and were limited to the following: quantity and quality of the influent wastewater, the bioreactor operating parameters or their combinations (Step A, Figure 1). The recorded results enabled the construction of a classification model for simulating the activated sludge sedimentation based on the sludge volumetric index, SVI. The binary system was selected, which indicates whether sludge bulking does (0) or does not occur (1).

Sedimentation is a complex phenomenon and the amount of data recorded at the test facility was limited; therefore, the variables which have a statistically significant impact on the investigated phenomenon were identified before the model for soft sensor could be constructed. The Fischer–Snedecor test was used for that purpose (Step B, Figure 1). The variables with numerical values of test probability over p = 0.05 were omitted from the model.

In Step C (Figure 1) the combinations of independent variables (x_i_) which are considered a basis for the construction of classification models, were created. In these analyses, the combinations of the influent wastewater quality and the bioreactor operating parameters were adopted. This issue is of importance to the feasibility of the control and monitoring of the processes taking place in a complex facility such as a WWTP, considering the numerous technical problems in its operation. Moreover, a lot depends on the function that it is supposed to satisfy. Importantly, the number of independent variables included in the model should not entail high-cost determinations of the wastewater quality indicators or the operating parameters of a reactor. Instead, it should be optimal for the applicable requirements and enable management of the facility.

In the next step of the analyses (Step D, Figure 1), the selected combinations of the independent variables of interest were assigned numerical values in the range 0–3, the sum of which within the respective variables (quality of wastewater and operating parameters) is standardized. The values of the adopted weight factors and the proposed standardizing functions describe the following:

- the number of independent variables x_i_ relating to the wastewater quality, duration of their determinations, and the bioreactor operating parameters: δ, λ;
- the cost of measurements of the wastewater quality indicators, bioreactor operating parameters (the value 0 relates to the lowest cost and 3 to the highest cost): f(δ), f(λ);
- the duration of measurement of the wastewater quality indicators (0 relates to those measurements in which the duration was lesser than one day, and 1 pertains to those lasting for more than one day): f(t);
- the possibility of using the selected model for the management, control or optimization of the WWTP functioning (the weight factor of 1 indicates that the model is applicable in the control, optimization, and the weight factor of 0 means that the model cannot be used for improving the efficiency of the WWTP): F(S).

In the next step (Step E, Figure 1), the combination of independent variables is used for creating classification models using different data mining methods. In the present analyses, before selecting a method the authors considered its complexity, the number of estimated coefficients within its structure, and the possibility of interpreting the results obtained. On the basis of results of such calculations, it is determined how they fit to those of the measurements—SPEC (determines the correctness of data classification in the set of data, including events when a activated sludge bulking occurred), SENS (determines the correctness of data classification in the set of data constituting cases when no activated sludge bulking occurred) [63,64,65].Using these results, the model class is found and, depending on the weight factor value, the calculation method is selected.

The established standardized weight values expressing the number of the independent variables that describe such parameters as the wastewater quality δ = f(z_1_, z_2_, …, z_q_), bioreactor operating parameters λ = f(f_1_, f_2_, …, f_g_), bacterial flora (e_1_, e_2_, …, e_t_), cost of analyses f(δ) and f(λ), duration of analyses f(t), and usefulness of the model for control and management of the bioreactor operation F(S), were used in determining the multi-dimensional weight factor vectors [δ f(δ) λ f(λ) f(t) F(S)]. They enabled the matrices to be found facilitating a fast and simple choice of the suitable data-mining method for identification of the activated sludge bulking (Step F, Figure 1).

When selecting a model, the proposed solution offers the possibility to introduce economic criteria to vary the independent variables that are included in the model (Step G, Figure 1). These independent variables relate to the number of data to be measured (volume and quality of the wastewater, and the reactor operating parameters) as well as to the possibility of using a specific model for the control, management and optimization of the WWTP operation.

The resulting soft sensor system for selecting the optimal data-mining method for the analysis of the activated sludge sedimentation can be applied in the daily operation of the WWTP and in its management, based on a variety of calculation methods, taking the wastewater volume and the bioreactor operating parameters into account.

### 2.3. Methods of Data Mining, Choice of Independent Variables and ConstructionCriteria for Soft Sensor

Taking into the account the issues with optimal selection of a method for the simulation of wastewater treatment plant processes, which was discussed in the introduction, the classification models were adopted for the analysis of the phenomenon considered. In order to determine the optimum between the accuracy and complexity of a model, as well as the number of independent variables, an analytical model—logical regression (LR)—was considered in the analyses. The model had the following form:

(1) $$p=\frac{exp\left(\sum_{j=1}^{k}\beta_{j}\cdot x_{j}+\beta_{0}\right)}{1+exp\left(\sum_{j=1}^{k}\beta_{j}\cdot x_{j}+\beta_{0}\right)}$$

in which the established β_j_ coefficients enable determining the impact of the input data (x_j_) on the probability of the occurrence of the phenomenon (p)—activated sludge bulking in this case.

Simultaneously, the application of models with increasing complexity was tested, starting from relatively simple ones (RF, BT) and finishing with a neural network methods like MLP and its subsequent modification, i.e., SVM. The mentioned methods, which were applied in this research work in creating the models for soft sensor predicting the activated sludge bulking, are highly efficient, as confirmed by a number of research works [32,49,62]. Taking into account the above, these models have been implemented in such statistical packages as R, STATISTICA, XLSTAT, SPSS, and can be used for building the models for predicting activated sludge bulking by many groups of users, including those responsible for the management of a WWTP.

In this elaboration, the statistical models were indicated by means of STATISTICA 12 software package. In order to devise models, the measurement data were divided into two sets: a learning set (75%) and a test set (25%). In the case of the logit model, based on the appropriate combinations of independent variables and on the assumed confidence interval of 0.05, the empirical coefficients were determined using formula (1). In the RF and BT model, assuming the number of trees up to 300, the structure of models was determined. In the MLP models, the number of neurons in the hidden layer was analyzed in the range (j–2·j + 1) (where: j—number of independent variables). In order to optimize the structure and weight values for the assumed number of neurons, different activation functions were analyzed, including: linear, exponential, sigmoidal, sinus, tangent—hyperbolic. For a complex number of neurons in the hidden layer and consecutively assumed activation functions, the fitting of calculation results to the measurements was determined. When the calculated values of SENS and SPEC for the assumed independent variables and the number of neurons as well as the activation function were minimal, the structure obtained was considered optimal. In the SVM method, Gaussian kernel was assumed, while the optimum values of C and γ were sought with the iteration method, substituting the values of above-mentioned parameters until the minimal SENS and SPEC values were achieved.

Given the fact that sludge bulking depends on the wastewater volume, its quality, operating parameters and the technology, the following general relationship can be formulated [11,62,65,66,67]:

(2) $$\{\begin{array}{c} SVI=f\left(Q,\;z_{1},\;z_{2},\;z_{3},\;\ldots z_{q},\;f_{1},f_{2},f_{3},\ldots f_{g},\;e_{1},\;e_{2},e_{3},\ldots ,e_{t}\right)\\ \;SVI\leq SVI_{lim}\;activated\;sludge\;does\;not\;bulk\;\\ \;SVI>SVI_{lim}\;activated\;sludge\;bulks\; \end{array}$$

where: z_1,2,3,q_—independent variables describing the quality of wastewater, f_1,2,3,g_—independent variables describing the bioreactor operating parameters, e_1,2,3,t_—independent variables describing the bacterial communities present in the activated sludge, and the technological solution of the bioreactor, q—the number of wastewater quality indicators, included in the mathematical model, g—the number of operating parameters of the reactor, included in the model, t—the number of independent variables, which describes the bacterial communities and has been included in the model.

Taking into account the complexity of the sedimentation process and the differences in the accessibility of the measured data within a WWTP, this paper provides detailed analyses to find the specific combination of data including the values of z_1,2,3,q_ and f_1,2,3,g_ for which the sludge bulking process can be identified. In the analyses, the variables e_1,2,3,t_ (Formula 2) were omitted. The influence of micaceous organisms, including filamentous bacteria, on the results of calculations of volumetric index of activated sludge was analyzed by Bezak-Mazur et al. [68]. Consequently, the following models were developed for the analyses:

(3) $$SVI_{q,g}=f\left(\;Q,z_{1},\;z_{2},\;z_{3},\;\ldots z_{q},\;f_{1},f_{2},f_{3},\ldots f_{g}\right)$$

(4) $$SVI_{q}=f\left(\;Q,z_{1},\;z_{2},\;z_{3},\;\ldots z_{q}\right)$$

(5) $$\;SVI_{g}=f\left(\;f_{1},f_{2},f_{3},\ldots f_{g}\right)$$

The model described by the relationship (4) includes the volume and indicators of the quality of the influent wastewater. Within the respective models, described by Equations (3)–(5), the authors sought such combinations of independent variables, including for instance the wastewater quality, of which the number is as small as possible and the results of simulation are consistent with the measured data:

(6) {1,2,3,…q, 1,2,3,…g}→min and SPEC(SENS)→max

(7) $$\left\{1,2,3,\ldots q\right\}\to \min\;and\;SPEC\left(SENS\right)\to maxSVI_{q}=f\left(\;Q,z_{1},\;z_{2},\;z_{3},\;\ldots z_{q}\right)$$

(8) {1,2,3,…g}→min and SPEC(SENS)→max

On the basis of the work [69] it was shown that for the Siktówka-Nowiny WWTP there is a general relationship:

(9) $$\{\begin{array}{c} SVI\left(t\right)=f\left(\;Q\left(t-1\right),\;BOD\left(t-1\right),TN\left(t-1\right),TP\left(t-1\right),\;MLSS\left(t-1\right),DO\left(t-1\right),\;m_{PIX}\left(t-1\right),T\left(t-1\right)\right)\\ \;SVI\leq 150\;activated\;sludge\;does\;not\;bulk\;\\ \;SVI>150\;activated\;sludge\;bulks \end{array}$$

where: (t)—values of independent variables measured at moment t; (t–1)—values of independent variables measured at moment (t–1).

On the basis of the relationship (9), it can be stated that the load of organics, nitrogen and phosphorus in the influent wastewater has a statistically significant effect on the sludge sedimentation in the test facility. The operating parameters of the reactor, such as the concentration of activated sludge, oxygen level, dosage of PIX, as well as temperature in the sludge chambers strongly correlate with the seasons of the year [33,62,69]–are important as well. After substituting the obtained relationships in Equations (3)–(5), the following was obtained:

(10) $$SVI\left(t\right)=f\left(Q\left(t-1\right),\;BOD\left(t-1\right),TN\left(t-1\right),TP\left(t-1\right),\;MLSS\left(t-1\right),DO\left(t-1\right),\;m_{PIX}\left(t-1\right),T\left(t-1\right)\right)$$

(11) SVI(t)=f(Q(t−1), BOD(t−1),TN(t−1),TP(t−1),T(t−1))

(12) $$SVI\left(t\right)=f\left(\;MLSS\left(t-1\right),DO\left(t-1\right),\;m_{PIX}\left(t-1\right),T\left(t-1\right)\right)$$

In the case of interest, for the Sitkówka-Nowiny WWTP, operated as an integrated system for the removal of carbon, nitrogen and phosphorus compounds, it was assumed that SVI_lim_ = 150 cm^3^/g, as reported in the literature [23].

### 2.4. Determining the Values of Weight Factors and Matrices of a Method Selection for Identification of Sludge Bulking

For a quantitative assessment of the differentiation of the adopted combinations of independent variables in predicting activated sludge sedimentation, as described above, the authors proposed an original system for determination of weight factors. The total effect of the aforementioned factors is expressed using the cumulative weight factor (W_tot_) the minimum value of which, taking into account the model-building criteria (mentioned below), can be calculated from the formula:

(13) $$W_{tot,min}=min\left\{min\left\{\delta ,f\left(\delta \right),f\left(t\right)\right\}+min\left\{\lambda ,f\left(\lambda \right)\right\}+F\left(S\right)\right\}$$

The general relationship (14) takes the following form:

(14) $$W_{tot}=\left\{\delta ,f\left(\delta \right),f\left(t\right)\right\}+\left\{\lambda ,f\left(\lambda \right)\right\}+F\left(S\right)$$

where: δ, f(δ), f(t), λ, F(λ), F(S)–according to the notation in the subsequent sections.

In practice, numerous variants of independent variables in models are encountered in the operation of a WWTP. This depends on the data obtained in measurements in the facility and on the requirements that a given model is expected to meet.

In Equation (13), the boundary conditions (π) facilitating the choice of independent variables for predicting SVI values were defined in parallel and in series:

- the condition determining the costs of determination and the number of indicators of the wastewater quality which are included in the model and reducibility of their value:
  (15) $$\pi_{1}:min\left\{\delta ,f\left(\delta \right),f\left(t\right)\right\}$$
- the condition determining the cost and number of measurements of the operating parameters which are included in the model and their reducibility:
  (16) $$\pi_{2}:min\left\{\lambda ,f\left(\lambda \right)\right\}$$
- the condition enabling minimization of the number and costs of measurements pertaining to the values of wastewater quality indicators and the reactor operating parameters:
  (17) $$\pi_{3}:min\left\{\delta ,f\left(\delta \right),f\left(t\right)\right\}+min\left\{\lambda ,f\left(\lambda \right)\right\}$$
- the condition minimizing the number and cost of measurements of the wastewater quality indicators and the reactor operating parameters so that the obtained model can be used in controlling the reactor operation:
  (18) $$\pi_{4}:min\left\{min\left\{\delta ,f\left(\delta \right),f\left(t\right)\right\}+min\left\{\lambda ,f\left(\lambda \right)\right\}+F\left(S\right)\right\}$$

In spite of the defined boundary conditions, the choice of the suitable data-mining method for the identification of activated sludge bulking and for the management of the WWTP operation is possible if the operating parameters of the bioreactor are included; this corresponds to the following relationships (16), (17), (18).

The data-mining method for simulating the volumetric index of activated sludge is selected after establishing independent variables in the model, according to the aforementioned calculation algorithm.

The function **δ** in Formula (14) describes the number of the quality indicators which were adopted in simulating the values of SVI. It takes the following form:

(19) $$\delta =\frac{\sum_{i=1}^{q}\left[z_{i}\right]}{\sum_{i=1}^{q}{\left[z_{i}\right]}_{max}}$$

where: z_i_ = 1, 2, …, q–number of the wastewater quality indicators included in the model; if the indicator is included in the model, then [z_i_] = 1, otherwise [z_i_] = 0, [z_i_]_max_—maximum weight of 1 indicating that the i-th indicator is included in the calculation model, in the case below q = Σ[z_i_]_max_ = 3.

The symbol **f(δ)** is the function of costs showing the impact of the cost of determining the quality indicators; it is described by the equation:

(20) $$f\left(\delta \right)=\frac{\sum_{i=1}^{q}\left[K(z_{i})\right]}{\sum_{i=1}^{q}{\left[K\left(z_{i}\right)\right]}_{max}}$$

where: K(z_i_)—weight of cost of measurement of the i-th indicator of the wastewater quality, included in the model; in this case, the number of indicators q = 3, and they are as follows: BOD, TN and TP. A literature survey [70,71] showed that the following relationship is true: K(TN) > K(TP) > K(BOD), where the value of TN is found by calculation Kim et al. [72]; therefore, the weight factors of K(TN) = 3 and K(TP) = 2 and K(BOD) = 1 were adopted in the calculations. For instance, if BOD and TP are included in the model, then the value of the function f(δ) = 1 + 2 + 0/(1 + 2 + 3) = 3/6. The proposed method takes into account the fact that the adopted weight factor values may be uncertain. In that case, it is acceptable to assume that the values K(z_i_) are stochastic and may be predicted with the Monte Carlo method.

The function f(t) in Formula (15) takes into account the duration of determination of the i-th indicator of the wastewater quality for simulating the values of SVI. It takes the values f(t,z_i_) = 1 for the time of determination longer than 24 h, otherwise, it is 0. In the model of interest, it was assumed that the values of the wastewater quality indicators in Equations (10), (11) may be modeled using statistical models [69,73,74,75]—for these cases, f(t) = 0.

The function **λ** describes the number of the bioreactor operating parameters adopted in calculating SVI. The function takes the following form:

(21) $$\lambda =\frac{\sum_{k=1}^{g}\left[z_{k}\right]}{\sum_{k=1}^{g}{\left[z_{k}\right]}_{max}}$$

where: z_k_ = 1, 2, …, g–number of the bioreactor operating parameters included in the model, if a parameter is included in the model, then [z_k_] = 1, otherwise, [z_k_] = 0, [z_k_]_max_—the maximum weight factor value of 1 indicates that the k-th operating parameter is included in the calculation model; in the case below, it is q = Σ[z_k_]_max_ = 3 (namely, DO, MLSS, T).

The symbol **f(λ)** is the function of costs, describing the impact of the cost of determining the quality indicators. It can be described by the following equation:

(22) $$f\left(\lambda \right)=\frac{\sum_{k=1}^{g}\left[K(z_{k})\right]}{\sum_{k=1}^{g}{\left[K\left(z_{k}\right)\right]}_{max}}$$

where: K(z_k_)—the weight factor of the measurement of the k-th bioreactor operating parameter, included in the model; in this case, the number of the indicators included is g = 3, and these are: DO, MLSS and T. Literature [61,70] has shown that the following relationship is true: K(MLSS) > K(DO) > K(T). Therefore, the following weight factors were adopted in the calculations: K(MLSS) = 3, and K(DO) = 2, and K(T) = 1. For instance, when the values MLSS and T are included in the model, then the value of the function is f(λ) = 1 + 3 + 0/(1 + 2 + 3) = 4/6.

Under operating conditions, the measured values from several hardware sensors are exposed to error, potentially affecting all the devices. Therefore, in sensitivity analyses, the recommended approach is to analyze the impact of several factors on the results of calculation. This can be described by the following equation:

(23) $$S\left(x_{1},x_{2},\ldots ,x_{j}\right)=\frac{\Delta p}{p_{0}\left(x_{1},x_{2},\ldots ,x_{j}\right)}=\frac{p\left(x_{1}+p\cdot \Delta x_{1},x_{2}+p_{1}\cdot \Delta x_{2},\ldots ,x_{j}+p_{j}\cdot \Delta x_{j}\right)}{p_{0}\left(x_{1},x_{2},\ldots ,x_{j}\right)}$$

Therefore, the function **F(S)** takes into account the fact that the selected independent variables in a model enable the control and adjustment of the SVI values. This can be described as follows:

(24) F(S)=f(λ,δ)

If the variables adopted in the calculations enable the control of the SVI values, then the function F(S) = 0, otherwise, F(S) = 1.

**Figure 2:**
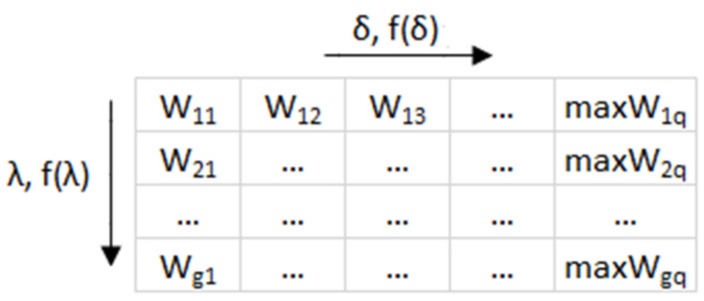
Matrix of the cases of interest and the weight factor values $W_{gq}$. where the following relationships are satisfied: λ = f(f_1_, f_2_, f_3_ …, f_g_) = f(λ), δ = f(z_1_, z_2_, z_3_ …, z_q_) = f(δ).

If the essential criteria for the model creation, encompassing the measured values showing the wastewater volume and quality and the bioreactor operating parameters are adopted, then a matrix of cases can be written (Figure 2) and the weight factor values W can be found from Equation (13). For the established combinations of independent variables in the matrix above and for the weight factor values W_tot_, the mathematical models for the identification of the activated sludge bulking are found using the selected data mining methods.

**Figure 3:**
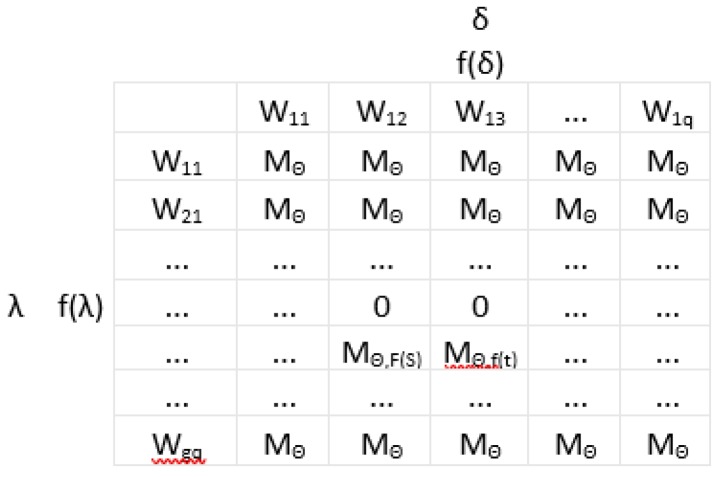
Matrices for the selection of methods for identification of sludge bulking.

The obtained results of simulation are expressed in the form of a matrix for the selection of methods (Figure 3). Such a matrix includes the models with the optimum fitting between the results of calculation and the measured results among all the methods considered.

In order to supplement the aforementioned methods and the matrices, additional criteria can be included which govern the developed model (controllability—M_Θ,F(S)_, duration of determination—M_Θ,f(t)_). A representative matrix for the selection of a method for the simulation of sludge bulking is shown in Figure 3. For M_Θ_ = 0, the selected method cannot be applied for assessing the capacity of sedimentation because independent variables were included in the model or due to the duration of determination of the wastewater quality indicators, which are included in the model. For instance, for M_Θ_ = M_Θ,F(S)_ = M_Θ,f(t)_ ≠ 0, the selected data-mining method can be used for predicting activated sludge bulking.

## 3. Results and Discussion

### 3.1. The Sludge Bulking Identification Method and the Choice of Independent Variables

On the basis of the results of measurements in the Sitkówka-Nowiny WWTP, the classification models for predicting SVI using the boosted tree (BT), random forest (RF), multilayer perceptron (MLP), support vector machines (SVM) and logistic regression (LR) methods were determined using the relationships (3)–(8), (11), (12), and (13). The variables A–G, which were statistically significant to the phenomenon of interest, were used in various combinations. Table 2 presents the values of measures of fit between the calculated and the measured results (SENS and SPEC).

The data in Table 2 indicate that the models found by means of the BT and RF methods are not over-learned. This is confirmed by the number of trees (N.T.) for the consecutive calculation variants (<300 trees). In the MLP method, the number of neurons is not greater than 2·j+1 (where: j–number of independent variables, included in the model). This seems to indicate that the obtained models are not over-learned [24]. In the hidden and output layers, usually the following activation functions are present: linear, exponential, tangent—hyperbolic, sinusoidal, and logistic.

The analysis of the data in Table 2 also indicates that the classification models for predicting SVI are characterized by different predictive powers. This is largely due to the choice of the independent variables which include the wastewater quality and the bioreactor operating parameters (or their combinations) as well as the selected methods (BT, RF, MLP, SVM, RL). The results of simulation have confirmed a significant effect of the content of biogenic compounds on the activated sludge sedimentation in the test facility. The parameter indicates that the phenomenon of activated sludge sedimentation is taking place [29].

The predictive power was observed to improve in the classification models after the inclusion of the various independent variables (BOD, TN, TP). Such a relationship can be perceived based on the model formed with the RF, BT and SVM methods. However, the approach where the wastewater quality indicators (A, B and C) and temperature in the activated sludge chambers were included, enables the management of the facility and application of the model for a soft sensor in optimizing the operation of the WWTP. These results were also confirmed in the studies of Pretorius and Rössle [76], who formulated the empirical relationships for predicting SVI from MLSS, T obtaining the value of R^2^ = 0.84. The above-mentioned relationship was also confirmed by the analyses of Szeląg and coauthors [69], who indicated the instrumental influence of the independent variables on sludge bulking by employing the logistic regression method. The possibility of employing logistic regression for sludge bulking identification was also shown by Bayo et al. [62], who presented the seasonal nature of the phenomenon, which confirms the influence of temperature on its course. An analysis of the examples of calculations for various combinations of independent variables A–G, indicates that the determination of a model with a high classification power does not require the inclusion of as many factors as the regression models. Such complex calculation algorithms are not necessary, either. Bagheri et al. [77] obtained a model of soft sensor characterized by a good fit with the measured data after including COD, TN, T, MLSS, DO, pH, TSS and modifying the MLP method in which the weight factors were corrected using a genetic algorithm. A similar approach to that of Bagheri et al. [77] was described by other researchers [26,56,57,78], focusing on the modification of an artificial neural network model in order to obtain better predictive power of their model. Interesting results of analyses were presented by Boztoprak et al. [25], who obtained reliable calculation results through observations with high-resolution cameras and the application of cellular neural networks (weights were optimized with genetic algorithms). Han and Qiao [78], based on the measurement results of only COD, TN, BOD, pH, DO and using modified MLP neural network models (hierarchical, hybrid– radial basis function (RBF) networks with clustering model (Kohonen maps) etc.), indicated the possibility of bulking simulation with a limited number of independent variables in comparison to the works of other authors [31,69,79].

Nonetheless, the use of such complex model structures may be problematic during the implementation in a WWTP facility. A comparison of the simulation results described by the aforementioned authors with the results of the studies indicates that there is no need to modify the methods used in the research work. The results of simulation, obtained with those methods for data relating only to the wastewater quality or only to the bioreactor operating parameters enable identification of the sludge bulking with high accuracy. This is confirmed by the obtained values of SPEC, SENS (Table 2). Looking at the obtained results in the aspect of reducibility of the number of independent variables, depending on the adopted method [24,69], one can conclude that using the classification model for the identification of sludge bulking and a rather limited number of independent variables, it is possible to obtain a good fit between the calculated and the measured results. This effect can be seen using non-modified methods (RF, BT, SVM, MLP, LR) and a considerable number of input data, including both the volume and quality of the wastewater and the bioreactor operating parameters. The aforementioned aspects were discussed in the work of Han and co-authors [26], and Han and Qiao [78], who compared the results of SVI value simulations obtained using modified models with those found by means of classic models (ARX, SVM, MLP), where the model structure was not corrected. It is also worth noting that the resulting soft sensor models can be used for the on-line control and adjustment of the activated sludge bulking. This allows improving the operation of the test facility and enables its real-time management. On the other hand, a compromise between the number of independent variables and accuracy of the soft sensor model is desirable. A large number of independent variables, as presented in the paper by Luo and Zhao [24]—Q, BOD, COD, TN, N-NH_4_, TP, TSS, T, MLSS, pH—or a higher number of measurable indicators of the wastewater quality, as described by Bagheri et al. [77], may lead to the problems with the continuity of independent variables and applicability of the soft sensor model in the management of a WWTP.

### 3.2. The Choice of a Sludge Bulking Identification Method for Various Independent Variables of Model

The data in Table 2 indicate that among the data-mining methods of interest in this paper, a high measure of fit (SPEC > 0.8 and SENS > 0.7) between the calculated and the measured results for SVI could be obtained based solely on the temperature in the activated sludge chambers, using a model built according to the RF and BT methods in the simulation. The results of calculations for the activated sludge simulation are improved by 10% after taking into account the values of wastewater quality indicators such as BOD, TN and TP in the model. The analysis results obtained are confirmed by the findings of Bagheri et al. [77] who indicated the influence of wastewater quality on the improvement of the prediction capabilities of the model (MLP) for the sludge bulking simulation. Similar dependences were also shown by Mirbagheri et al. [79], who investigated the influence of independent variables (wastewater quality, operational parameters) on the results of COD and TP simulations at the wastewater treatment plant discharge.

As an alternative to BT, the SVM algorithm can also be used for sludge bulking identification, except that this is one of the most complex models of all those considered in this research work. It is also worth noting that among all the employed methods, the BT method enables the prediction of SVI from indicators of the amount and quality of the influent wastewater. This is possible knowing just the value of total nitrogen. Inclusion of the other independent variables, such as TN, TP, one by one enables conducting the simulation calculations for the activated sludge bulking with high accuracy (SENS, SPEC > 0.80). Even though the sedimentation capacity of the sludge can be identified based on the measured temperature, amount and concentration of contaminants in the influent wastewater, these values cannot be used for adjusting the bioreactor operating parameters. In the aspect of operation and reliability of a WWTP, these models are applicable when the influent conditions do not indicate any possibility of activated sludge bulking. Similar dependences were obtained by Bayo et al. [62], who indicated the possibility of the sludge bulking process identification based on the knowledge of weather conditions, omitting the operational parameters of the bioreactor. Such a case and an analysis of the condition of activated sludge in the aspect of its bulking are only possible for a linear combination of independent variables, including the amount and quality of the wastewater, and weather conditions [62]. However, for the aforementioned data, the possibility of optimal adjustment of the bioreactor settings is limited, and this affects the operation of the facility.

From the simulation calculations performed, it can be observed that among the bioreactor operating parameters of interest (MLSS, DO), which enable adjustment of the SVI values, the lowest predicting errors are obtained for the model determined from MLSS. The aforementioned dependence is confirmed by the analyses conducted by Comas et al. [29], who devised an expert system for the assessment of the impact and investigating the interaction between the wastewater quality at the inlet and outlet, operational parameters and activated sludge bulking. In that case, a satisfactory fit (SPEC = 0.96 and SENS = 0.87) between the calculated and the measured values is obtained by means of the BT method. Lower values of error were obtained by MLP (SENS by 4% and SPEC by 22.67%) as well as SVM (SENS by 3.12% and SPEC by 17.95%). Reduced measurement errors (SENS, SPEC values) were obtained in the models, taking into account the sludge temperature. Therefore, for the variables MLSS, T, satisfactory results of calculations were obtained by means of BT, RF, SVM, MLP and LR. Under the operating conditions, the soft sensor model based on logistic regression is the simplest and easiest to implement. In the case of landslide identification, a good fit between the results of calculations obtained with a logit model was achieved, in relation to more complex statistical methods (SVM) [80]. Similar results were obtained through the analysis of methodical data, while comparing the results of the analyses conducted with the ANN method [81]. The calculation of SVI from DO alone provided satisfactory results with BT, RF, as confirmed by the values of SPEC >0.9 and SENS of at least 0.7. In the case of a combination of independent variables including DO and T, a good fit between the calculated and the measured data was obtained with BT, RF as well as by means of MLP and SVM, which are more complicated methods. A compilation of the aforementioned independent variables (which are the controlling variables since they enable adjustment of SVI, namely MLSS, DO and T), enables the activated sludge bulking identification using the methods based on neural networks (MLP, SVM), developed from the regression tree concept, that is BT and RF, as well as using them for monitoring and for the control of technological processes within a WWTP. The results obtained show that inclusion of the values of wastewater quality indicators (BOD, TN,TP) in the calculation model SVI = f(DO, T) or SVI = f(DO) provides lower errors since SENS, SPEC are higher. Among the methods considered, the lowest values of errors were obtained for the RF and BT methods in most cases. Using the SVM method, good measures of fit between the measured and the simulated data were obtained for the variables including the values of DO, T, indicators of amount and quality—BOD, TN. Among the classification models discussed in this paper (Table 2), the best predictive power was shown by those in which the values of wastewater quality indicators as well as the bioreactor operating parameters were included. For that combination of independent variables, the results of simulation are characterized by a good fit with the measured values for those methods in which the soft sensor model structure is not very complex, the number of calibrated parameters is small (RF, BT, LR) as well as in more complex methods (SVM, MLP). Significant similarity of the results pertaining to the calculations of technological wastewater treatment plant parameters (energy consumption, biogas production) obtained with the RF and MLP methods was confirmed by the analyses performed by Kusiak et al. [58,82]. Among the combinations considered in this paper, the best results of calculations were obtained for the combination involving Q, TN, TP, MLSS, DO, T, m_PIX_. On the one hand, these data contribute the information on the number of compounds that the microorganisms in the bioreactor are provided with and, on the other, they describe the dynamics of the processes taking place in the activated sludge. In this approach, the variables are supplemented with the coagulant dosage (it enables the adjustment of SVI and of bulking so that the values of MLSS and DO can be kept within the optimal range). The proposed approach offers the possibility of choosing the optimal settings in the operation of a WWTP and improving the sedimentation capacity of the activated sludge, even though the values of MLSS, DO may not guarantee SVI below 150 cm^3^/g.

Moreover, the data in Table 2 show that the activated sludge bulking can also be identified for a slightly reduced range of independent variables as well as for other combinations, and the results of calculations will also fit well to the measured values (BOD/TP, BOD/TN, DO, MLSS, T, m_PIX_). For instance, the logit model for a combination of independent variables relating to the amount and quality of wastewater and the bioreactor operating parameters is described by the following relationship:

(25) $$X=28.24-0.68\cdot T-2.66\cdot MLSS-1.82\cdot DO-0.56\cdot m_{PIX}+0.0008\cdot Q\cdot TN+0.0009\cdot Q\cdot TP+0.0001\cdot Q\cdot TP$$

where: L_TN_—total nitrogen in the incoming wastewater, L_TP_—total phosphorus in the influent wastewater, L_BOD_—biochemical oxygen demand of the influent wastewater. The model above comprises the following variables having a random effect on the sludge bulking:

(26) $$\omega =0.0008\cdot L_{TN}+0.0009\cdot L_{TP}+0.0001\cdot L_{BOD}$$

and the controlling variables:

(27) $$\tau =28.24-0.68\cdot T-2.66\cdot MLSS-1.82\cdot DO-0.56\cdot m_{PIX}$$

where: τ, ω, L_TP_, L_TN_, L_BOD_, MLSS, DO, T, m_PIX_–independent variables, marked as above.

### 3.3. Impact of Uncertainty of Measured Data on Activated Sludge Bulking Identification

**Figure 4:**
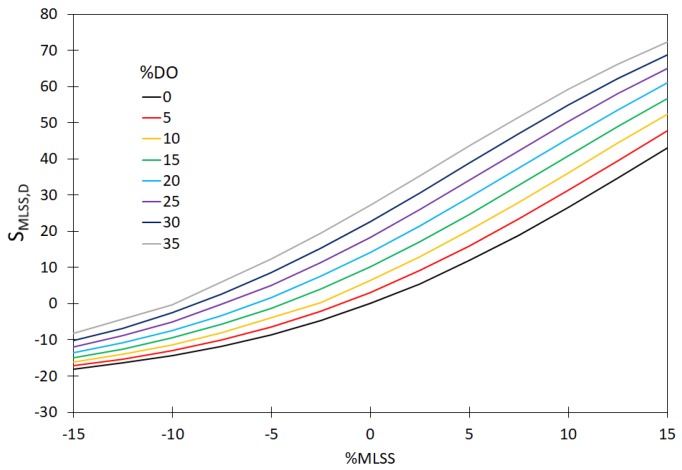
The effect of variation of the MLSS and DO values on the sensitivity coefficient S_MLSS,DO_.

**Figure 5:**
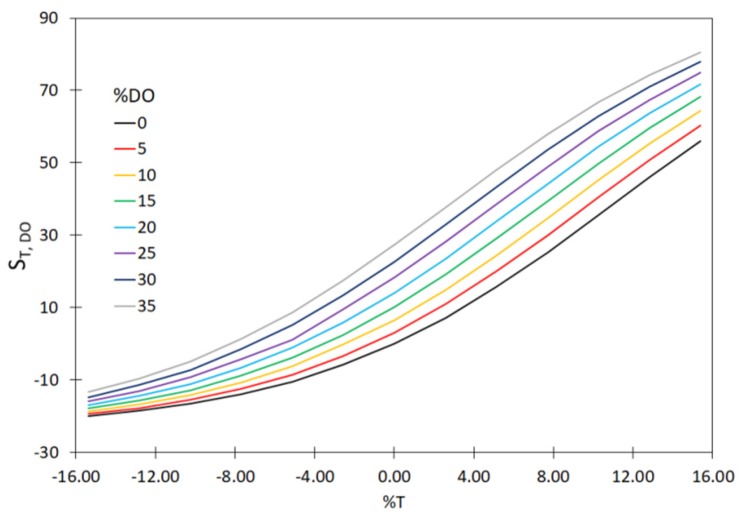
The effect of variation of the T and DO values on the sensitivity coefficient S_T,DO._

**Figure 6:**
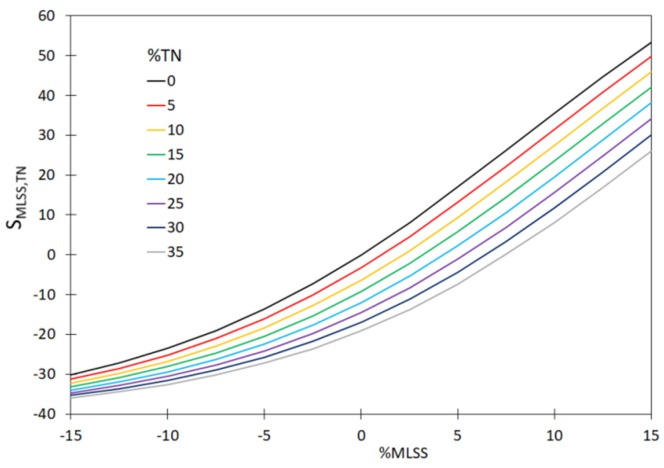
The effect of variation of the MLSS and TN values on the sensitivity coefficient S_MLSS,TN._

**Figure 7:**
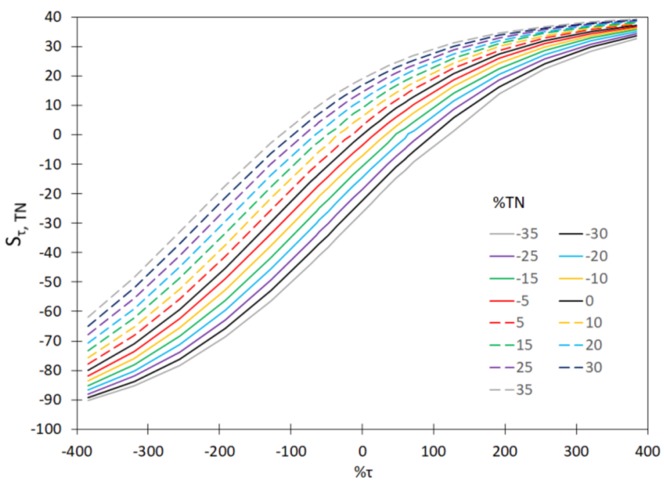
The effect of variation of the τ and TN values on the sensitivity coefficient S_τ, TN_.

In order to demonstrate that it is necessary to continually calibrate the hardware sensors installed in the WWTP for measuring T, MLSS, DO and TN, the values of sensitivity coefficients (S_xj_, %) were determined. Those calculations included the measurement errors for a single hardware sensor and for two and three hardware sensors. The measured values of the sensitivity coefficients, based on the relationship (23) are shown in Figure 4, Figure 5 and Figure 6. Moreover, the analyses included the determination of variability of the sensitivity coefficient vs. measurement error for the values MLSS, DO, TN; (Figure 7). The measurement errors for MLSS, DO were expressed as the coefficient τ, described by Equation (27).

The plotted curves in the figures below indicate that the accuracy of the measurement of MLSS, DO, TN has a significant effect on the values of the sensitivity coefficients—S_MLSS,DO_ (Figure 4) as well as S_T,DO_ (Figure 5), S_MLSS,TN_ (Figure 6) and S_τ,TN_ (Figure 7). The curves in Figure 4, Figure 5, Figure 6 and Figure 7 indicate that a significant impact on the uncertainty of identification and on the monitoring of the sedimentation capacity of the activated sludge is particularly attributable to the errors resulting in the over-estimation of real values (theoretical ones, not affected by the measurement errors). In particular, this is visible if two or three hardware sensors fail at a time. It is also worth noting that the analyses described by other authors in previous reports only related to the single hardware sensors. The curves obtained (Figure 4, Figure 5 and Figure 6) show that the proposed approach enables the analyses of a bioreactor operation in a wider range than before, should the measured data be uncertain. Thus the results obtained are confirmed in the analyses performed by Comas et al. [29], who indicated a diversified influence of bioreactor operational parameters (MLSS, DO) and the quality of wastewater at the inlet (BOD, TN) on the activated sludge bulking by employing the fuzzy set method. The aforementioned relation is also confirmed by the analyses of Bagheria et al. [77], who identified the influence of the analyzed independent variables describing the wastewater quality (COD, TN, TSS) and operational parameters (T, MLSS, DO, pH) on the activated sludge bulking.

This is the condition of the optimal choice of settings for the bioreactor, to guarantee the desirable sedimentation capacities (SVI <150 cm^3^/g) and high reliability of the operation within the entire WWTP. These two factors are directly connected with the correct management of the WWTP operation, resulting in unhindered sludge dewatering and providing the optimal values of quality indicators of the effluent wastewater.

### 3.4. Matrices of the Choice of a Sludge Bulking Identification Method

From the weight factor values, established for the wastewater quality indicators (BOD—1, TN—3, TP—2) and for the bioreactor operating parameters (MLSS—3, DO—2, T—1), respectively, the numerical values for the functions f(λ) and f(δ) were determined. On this basis, the vectors [f(λ) f(δ)] were determined; then, it was established which of the considered methods should be used to make the soft sensor model useful for the purposes of calculations and predicting the sludge bulking, without taking into account any aspects concerning the process control and management (Table 3 and Table 4). The models for which the established values of the measures of fit for SPEC, SENS were the highest among all of those considered in this paper were regarded as the dedicated soft sensor models. Table 3 shows those methods for which the obtained values of the measures of fit were the maximum. Table 4 shows the applicability of the logistic regression LR (as the only one among the methods considered in which the chosen model is in the form of an empirical equation) for the sludge bulking simulation for the value [f(λ) f(δ)]. The same table comprises the cases for which the established values of SENS, SPEC >0.8 (a very good model—VG) and SENS, SPEC >0.9 (an excellent model). In the other cases of calculations, where one of the above-mentioned conditions was not met, the sludge bulking identification methods, referred to in Table 3 were adopted.

On the basis of the matrices, determined in Table 3, it is possible to choose the suitable method without having to perform the simulation calculations with various methods, taking into consideration the measured data that are available from the WWTP and that include the following: quality indicators (δ), their costs expressed as the function f(δ), the bioreactor operating parameters (λ) and their costs—f(λ). Moreover, to simplify the method selection with regard to the number of independent variables in the system: wastewater quality indicator–reactor operating parameters (1–1, 2–2, 3–3), the typical regions for such combinations were determined, which also helps choosing the bulking simulation method. Other variants (1–2, 2–1, 2–3, etc.) depend on the local conditions and data recorded within the specific WWTP. The proposed approach enables diagnosing and supervising the activated sludge bulking in the continuous system, which directly affects the input data (independent variables) included in the model. Moreover, the results in Table 3 and Table 4 indicate that—in many cases—logistic regression is an alternative to complex calculation models. The model obtained is the empirical equation in which the established coefficients can be used for determining the effect of selected independent variables on the activated sludge sedimentation process.

The other methods (BT, RF, MLP, SVM) require performing additional calculations. The matrices obtained and described above (Table 3, Table 4) enable the methods to be matched to simulation with respect to the number of the wastewater quality indicators, the bioreactor operating parameters, and the costs of their determination. Nonetheless, taking into consideration the operation of the wastewater treatment facilities, the soft sensor models which can be applied and which are potentially useful in the monitoring and supervision of the wastewater treatment processes are of the greatest importance. Therefore, using the developed algorithm and introducing a limit of the duration of determination indicators—f(t), a matrix of cases was obtained and is shown in Table 5.

The resulting matrix is an extended version of the matrix shown in Table 3; however, the variants included in it can be used for monitoring the operation of a WWTP, for its control, as well as for the adjustment of the bioreactor settings. The number of feasible variants results from the fact that the BOD determination is a time-consuming process, which affects the applicability of the model. However, assuming that the value of the parameter or that of the other selected wastewater quality indicators can be modeled by means of a statistical model (value of the function f(t) = 1), every case needs to be analyzed individually. The cost estimation is made even more complex by the fact that the costs of analysis pertaining to the wastewater quality indicators need to be assessed on a case-by-case basis. The reason is that it is very difficult to establish independent variables describing the values of selected wastewater quality indicators taking into account the local conditions.

## 4. Conclusions

The results of the analyses presented above show that the soft sensor model developed based on the selection of a data-mining method for sludge bulking simulation takes into account both the number of the wastewater quality indicators and the bioreactor operating parameters, as well as the costs of their determination. The reliability of the input data for the soft sensor model is an important criterion, as shown by a detailed analysis of sensitivity which enabled an assessment of the effect of one, two or three independent variable(s) on the results of calculation of the activated sludge bulking.

The simulations indicate that the procedure of soft sensor development described in this paper helps in choosing the calculation methods (RF, BT, SVM, MLP, LR) for the sludge bulking simulation, while minimizing the costs of measurements of such parameters as: wastewater quality indicators, the bioreactor operating parameters, duration of determinations, and the number of the independent variables included in the soft sensor model. The calculations show that a suitably selected simulation method enables the activated sludge bulking to be predicted with high accuracy from the load of contaminants arriving in the WWTP with the influent wastewater or the bioreactor operating parameters, and the combinations thereof.

The simulations and the matrices of the cases obtained and described above seem to indicate that the management of a WWTP in the aspects of its control, adjustment of settings and monitoring of activated sludge bulking can be performed by means of complex calculation methods such as neural networks and their modifications, having a simpler structure of mathematical algorithms such as logistic regression. The proposed soft sensor model enables on-line diagnosis in the operation of a WWTP because it includes various conditions potentially encountered in the course of a WWTP operation (failure of the wastewater quality hardware sensors, lack of continuity of measurements, errors in measuring the operating parameters, etc.).

The selection of the data-mining method depends on the user, although it is governed by the availability, the commercial attractiveness of the available software for modeling the operation of WWTP, and how the given software is operated. The soft sensor models for predicting the activated sludge bulking by means of the methods proposed in this paper (RF, BT, SVM, MLP, LR) can be built using the generally available statistical models, which are accessible to many groups of users.

## Figures and Tables

**Table 1:** Results of measurements of the volume and quality of wastewater and the reactor operating parameters in the Sitkówka-Nowiny wastewater treatment plant (WWTP).

Indicators	Units	Winter				Spring-Fall			
		Min	Mean	Max	Standard Deviation	Min	Mean	Max	Standard Deviation
Q	m^3^/d	29,952	39,364	88,986	6563	30,125	41,842	94,772	8559
BOD	mgO_2_/l	151	290	489	81.83	132	340	557	81.2
COD	mg O_2_/l	384	782	1183	161.4	342	820	1703	178.2
TSS	mg/l	136	315	474	62.76	110	350	572	89.4
N-NH_4_	mg/l	28	48.9	62	5.68	22	54.52	66.9	7.13
TN	mg/l	56.2	82.01	95.16	8.42	39.9	95.15	124.1	11.58
TP	mg/l	3.1	7.22	12.1	1.44	3.5	7.83	12.6	1.65
T	°C	10	11.9	13.5	0.8	11.3	17.8	23	3.1
DO	mg/l	1.8	2.85	3.25	0.8	1.51	2.2	3.25	0.65
MLSS	mg/l	2.85	4.95	6.54	0.84	2.15	4.11	5.28	0.95
MLSS_R_	mg/l	6.62	8.7	14.92	0.72	5.03	7.81	11.86	0.1
WAS	t.s.m/d	12.69	15.35	18.35	3.51	10.02	12.35	17.25	3.77
RAS	%	85.2	102.9	152	16.25	75.2	83.06	120.5	24.4
SVI	cm^3^/g	154	198	291	35	90	138	200	37
m_PIX_	m^3^/d	0	0.81	1.75	0.27	0	0.84	1.82	0.28

where: Q—inflow to wastewater treatment plant, BOD, COD—biochemical/chemical oxygen demand, TSS—total suspended solids, N–NH4—ammonium nitrogen, TN—total nitrogen, TP—total phosphorus, T—temperature in the activated sludge chambers, DO—oxygen concentration in the bioreactor, MLSS—concentration of activated sludge, MLSSR—concentration of return sludge, WAS—excess amount of sludge, RAS—degree of recirculation, mPIX—dosage of PIX, SVI—sludge volumetric index.

**Table 2:** List of values of the measures of fit between the calculated and the measured results (testing data) (SENS– sensitivity, SPEC–specifity) for SVI, obtained with various methods for a combination of independent variables (quantity and quality of wastewater, bioreactor operating parameters).

Independent	BT			LR		MLP			SVM			RF		
	SP	SE	N.T.	SP	SE	SP	SE	N:H:E	SP	SE	C, γ	SP	SE	N.T.
A	0.65	0.81	19	0.27	0.88	0.42	0.87	5:exp,lin	0.38	0.92	900,0.35	0.46	0.88	170
B	0.88	0.81	88	0.35	0.94	0.31	0.96	4: sin,lin	0.31	0.98	100,0.40	0.35	0.88	60
C	0.62	0.83	19	0.27	0.92	0.46	0.88	3: lin,lin	0.50	0.85	1000,0.25	0.46	0.88	20
A,B	0.85	0.83	19	0.38	0.90	0.48	0.92	8:exp,tanh	0.42	0.98	800; 0.35	0.00	1.00	25
A/B	0.77	0.73	11	0.27	0.85	0.35	0.94	3: tanh, lin	0.54	0.85	800;0.25	0.58	0.81	40
A/C	0.73	0.75	38	0.35	0.85	0.35	0.92	3:sin,lin	0.50	0.90	800;0.35	0.69	0.79	40
A,C	0.65	0.75	11	0.27	0.85	0.31	0.94	3:tanh,lin	0.54	0.87	800;0.80	0.58	0.79	40
D	0.81	0.88	183	0.65	0.87	0.73	0.85	3:exp, sin	0.62	0.88	900;0.33	0.88	0.77	30
D,A/B	0.88	0.77	10	0.68	0.87	0.65	0.87	4:lin,exp	0.73	0.85	800;0.35	0.88	0.81	40
D,A/C	0.88	0.88	155	0.68	0.88	0.69	0.85	3:lin,lin	0.73	0.87	800;0.33	0.88	0.80	30
D,B	0.88	0.77	10	0.62	0.88	0.65	0.90	7:tanh,lin	0.73	0.87	900;0.33	0.88	0.82	10
D,B,C	0.88	0.80	24	0.62	0.88	0.68	0.90	6:tanh,lin	0.76	0.88	800;0.25	0.88	0.86	30
D,A,B,C	0.92	0.83	14	0.65	0.92	0.69	0.94	9:lin,sin	0.77	0.90	1000;0.25	0.85	0.87	30
E	0.88	0.75	119	0.62	0.83	0.88	0.92	5:log,exp	0.85	0.88	900;0.40	0.50	0.98	119
E,D	0.95	0.96	98	0.88	0.96	0.92	1.00	5:exp,lin	1.00	0.98	900;0.25	0.88	0.92	30
E,F	0.96	0.87	98	0.65	0.90	0.88	0.96	5:lin,lin	0.88	0.96	900;0.20	0.46	1.00	200
F	0.92	0.81	195	0.00	1.00	0.46	0.90	4:tanh,tanh	0.58	0.85	900;0.50	0.92	0.71	30
D,F	0.88	0.77	15	0.62	0.88	0.73	0.85	7:log,lin	0.65	0.88	700;0.33	0.88	0.77	30
D,F,B	0.92	0.90	15	0.65	0.90	0.69	0.90	7:exp, lin	0.69	0.90	700;0.25	0.85	0.85	30
D,F,A/B	0.90	0.84	5	0.70	0.88	0.85	0.79	6:tanh,lin	0.81	0.87	900;0.33	0.88	0.77	30
D,F,B,C	0.88	0.81	10	0.65	0.90	0.69	0.92	8:tanh,tanh	0.81	0.88	800;0.25	0.88	0.85	30
F,B	0.85	0.88	114	0.35	0.92	0.50	0.87	3:tanh,tanh	0.46	0.96	700;0.20	0.88	0.77	140
F,B,C	0.92	0.92	186	0.31	0.94	0.55	0.96	5:sin,lin	0.54	0.85	500;0.35	0.54	0.85	20
F,C	0.85	0.73	15	0.23	0.90	0.50	0.88	3:lin,lin	0.54	0.87	600;0.40	0.50	0.75	30
D,F,E	1.00	0.93	162	0.88	0.96	0.96	0.98	7:exp,lin	0.96	0.98	600;0.25	0.08	1.00	30
A/C,F,E	1.00	0.88	119	0.69	0.90	0.88	0.94	7:lin,lin	0.88	0.94	600;0.35	0.85	1.00	30
A/C,F,E,D	1.00	0.96	195	0.92	0.96	0.91	0.95	7: lin,sin	0.93	1.00	600;0.20	0.46	1.00	60
A/C,A/B,F,E,D	1.00	0.97	100	0.94	0.96	0.92	1.00	3:tanh,exp	0.94	0.98	200;0.33	0.88	1.00	100
B,C,E,F,D	1.00	0.96	119	0.96	0.94	0.95	1.00	8:tanh,lin	0.88	0.98	100;0.17	0.81	1.00	70
B,C,E,F,D,G	1.00	0.98	195	0.96	0.98	0.99	1.00	4: lin,lin	0.92	0.98	100;0.25	0.88	0.96	70
B,C,E,F	0.96	0.88	195	0.85	0.94	0.93	0.99	4: lin,lin	0.69	0.96	200;0.35	0.66	0.91	70
B,E,F,D,G	1.00	0.96	195	0.85	0.94	0.95	1.00	6:lin,tanh	0.92	0.98	50;0.25	0.85	0.92	30
B,E,F,D	1.00	0.94	195	0.96	0.96	0.97	1.00	5: tanh,log	0.92	0.95	70;0.20	0.73	1.00	30
A/C,A/B,F,E,D,G	1.00	0.98	195	0.97	0.96	1.00	0.98	9:exp,lin	0.98	0.99	100;0.14	0.93	1.00	30
A/C,A/B,F,E,G	1.00	0.90	119	0.73	0.88	0.81	0.96	4:lin,lin	0.88	0.94	900;0.25	0.85	0.96	170
F,E,G	0.96	0.88	119	0.65	0.90	0.78	0.96	8:sin,exp	0.81	0.94	700;0.35	0.77	0.94	30
F,E,G,D	1.00	0.96	199	0.96	0.96	0.96	0.96	7:exp,lin	1.00	0.94	700;0.2	0.81	1.00	40

where: A—BOD, B—TN, C—TP, D—T, E—MLSS, F—DO, G—m_PIX_, SP—SPEC, SE—SENS.

**Table 3:** A matrix of cases showing relationships between the values of the functions f(δ), f(λ) and the classification models for predicting activated sludge bulking.

δ/λ		0	1/3	1/3	1/3 (2/3)	2/3	2/3	3/3
	f(δ)/f(λ)	0	1/6	2/6	3/6	4/6	5/6	6/6
**0**	**0**	X	BT	BT	BT	BT	BT	BT
**1/3**	**1/6**	RF	BT	RF	RF	RF	RF	BT
**1/3**	**2/6**	BT	MLP	BT	BT	BT	BT	BT
**1/3(2/3)**	**3/6**	BT	BT	MLP	BT	BT	BT	MLP
**2/3**	**4/6**	SVM	BT	MLP	SVM	BT	MLP	BT
**2/3**	**5/6**	SVM	MLP	MLP	BT	MLP	MLP	BT
**3/3**	**6/6**	SVM	SVM	MLP	BT	SVM	BT	BT

**Table 4:** A matrix of cases showing relationships between the values of the functions f(δ), f(λ) and the classification models, with a particular focus on the logit for predicting activated sludge bulking.

δ/λ		0	1/3	1/3	1/3 (2/3)	2/3	2/3	3/3
	f(δ)/f(λ)	0	1/6	2/6	3/6	4/6	5/6	6/6
**0**	**0**	X	BT	BT	BT	BT	BT	BT
**1/3**	**1/6**	RF	BT	RF	RF	RF	RF	BT
**1/3**	**2/6**	BT	MLP	BT	BT	BT	BT	BT
**1/3(2/3)**	**3/6**	LRVG	LRVG	LRVG	LRVG	LRVG	LRVG	LRVG
**2/3**	**4/6**	LRVG	LREX	LREX	LREX	LREX	LREX	LREX
**2/3**	**5/6**	SVM	LRVG	LRVG	LRVG	LRVG	LRVG	LREX
**3/3**	**6/6**	LRVG	SVM	MLP	BT	SVM	LREX	LREX

where: VG—the logit model, where SPEC, SENS >0.8, EX—the logit model, where SPEC, SENS >0.9.

**Table 5:** A matrix of cases showing the relationships between the values of the functions f(δ), f(λ), f(t) and the classification soft sensor models for predicting the activated sludge bulking.

f(t)		0	1	0	0	1	0	1
δ/λ		0	1/3	1/3	1/3 (2/3)	2/3	2/3	3/3
	f(δ)/f(λ)	0	1/6	2/6	3/6	4/6	5/6	6/6
**0**	**0**	X	X	BT	BT	X	BT	X
**1/3**	**1/6**	RF	X	RF	RF	X	RF	X
**1/3**	**2/6**	BT	X	BT	BT	X	BT	X
**1/3(2/3)**	**3/6**	BT	X	MLP	BT	X	BT	X
**2/3**	**4/6**	SVM	X	MLP	SVM	X	MLP	X
**2/3**	**5/6**	SVM	X	MLP	BT	X	MLP	X
**3/3**	**6/6**	SVM	X	MLP	BT	X	BT	X
